# An interpretive review of consensus statements on clinical guideline development and their application in the field of traditional and complementary medicine

**DOI:** 10.1186/s12906-017-1613-7

**Published:** 2017-02-17

**Authors:** Jennifer Hunter, Matthew Leach, Lesley Braun, Alan Bensoussan

**Affiliations:** 1 NICM, University of Western Sydney, Locked Bag 1797, Penrith, NSW 2751 Australia; 2 Menzies Centre for Health Policy, School of Medicine, University of Sydney, Sydney, Australia; 3 Australian Research Centre in Complementary & Integrative Medicine, University of Technology Sydney, Sydney, Australia; 4 Blackmores Institute, Sydney, Australia; 5 Monash/Alfred Psychiatric Research Centre, Monash University, Melbourne, Australia

**Keywords:** Evidence based medicine, Guideline, Practice guideline, Complementary medicine, Integrative medicine, Literature review

## Abstract

**Background:**

Despite ongoing consumer demand and an emerging scientific evidence-base for traditional and complementary medicine (T&CM), there remains a paucity of reliable information in standard clinical guidelines about their use. Often T&CM interventions are not mentioned, or the recommendations arising from these guidelines are unhelpful to end-users (i.e. patients, practitioners and policy makers). Insufficient evidence of efficacy may be a contributing factor; however, often informative recommendations could still be made by drawing on relevant information from other avenues. In light of this, the aim of this research was to review national and internationally endorsed consensus statements for clinical guideline developers, and to interpret how to apply these methods when making recommendations regarding the use of T&CM.

**Method:**

The critical interpretive review method was used to identify and appraise relevant consensus statements published between 1995 and 2015. The statements were identified using a purposive sampling technique until data saturation was reached. The most recent edition of a statement was included in the analysis. The content, scope and themes of the statements were compared and interpreted within the context of the T&CM setting; including history, regulation, use, emerging scientific evidence-base and existing guidelines.

**Results:**

Eight consensus statements were included in the interpretive review. Searching stopped at this stage as no new major themes were identified. The five themes relevant to the challenges of developing T&CM guidelines were: (1) framing the question; (2) the limitations of using an evidence hierarchy; (3) strategies for dealing with insufficient, high quality evidence; (4) the importance of qualifying a recommendation; and (5) the need for structured consensus development.

**Conclusion:**

Evidence regarding safety, efficacy and cost effectiveness are not the only information required to make recommendations for clinical guidelines. Modifying factors such as burden of disease, magnitude of effect, current use, demand, equity and ease of integration should also be considered. Uptake of the recommendations arising from this review are expected to result in the development of higher quality clinical guidelines that offer greater assistance to those seeking answers about the appropriate use of T&CM.

## Background

Traditional and complementary medicine (T&CM) refers to a conglomerate of health-related interventions and therapies not usually considered mainstream by the Western medical system. T&CM includes (but is not limited to) naturopathy, traditional Chinese medicine, Ayurvedic medicine, homeopathy, chiropractic, osteopathy, massage therapy, yoga and meditation. In such a multifarious field with divergent training requirements, different models of regulation, and myriad treatment options informed by varying (and sometimes inconsistent) evidence, it is not surprising there is considerable diversity in clinical practice [1]. The impact of these inconsistent practices on patient outcomes, patient satisfaction and professional credibility can be significant [2].

Clinical guidelines are *“systematically developed statements to assist practitioner and patient decisions about appropriate health care for specific clinical circumstances”* [3] that aim to reduce unnecessary variations in service delivery by informing a rational approach to the management of patients, as well as guiding healthcare policies. Evidence-based clinical guidelines were initially almost solely based on evidence of efficacy and safety [4–6]. The limitation of this approach was that it ignored other important considerations when developing guidelines to meet the healthcare needs of a population [7]. Increasingly, the importance of contextual information and qualifying statements about the burden of disease, economic impact, current use, patient values and preferences and equity, and the need for transparency throughout the development process have been adopted as guideline development standards [8–10]. Despite these standards, it is not uncommon for clinical guidelines and health policies regarding T&CM to only consider the evidence for safety, efficacy and cost-effectiveness, if they are considered at all [11].

The quality of clinical guidelines continues to be a matter of concern, hence the development of various guideline appraisal tools such as the AGREE II [12, 13]. In the field of T&CM, standard medical guidelines are fraught with inconsistencies and unhelpful recommendations. For example, reviews of guidelines endorsed by the UK National Institute for Health Care and Excellence (NICE) or the Scottish Intercollegiate Guidelines Network (SIGN) have found that many lacked transparency and consistency about the inclusion or exclusion of T&CM [14–16]. The conclusions drawn from the available evidence often overestimated or underestimated potential benefits. In many instances, even when one or more T&CM interventions were reviewed by the guideline developers, either no recommendations or nonspecific recommendations, such as ‘practitioners should discuss T&CM use with their patients’ or ‘more research is needed’ were made. General statements provide little guidance for clinical decision making and could be viewed as ‘holding statements’ rather than serving any real purpose.

Given the aforementioned findings, clinical guidelines of higher quality are urgently required to guide the safe and rational use of T&CM in practice [17]. Indeed, there are many instances where specific recommendations are often needed in T&CM practice and policy. As Table 1 illustrates, the decision to appraise an intervention or otherwise in a guideline is not always (nor should it be) dependent on data from clinical trials.

**Table 1:** Instances where T&CM should be considered in guideline development

1. The potential for at least modest clinical benefit where there is a large burden of disease^a^;
2. High quality traditional evidence about safety and effectiveness that address a continuing burden of disease;
3. Cost-effective interventions that address a continuing burden of disease^a^;
4. The potential for catastrophic risks from the T&CM intervention or its alternative;
5. Unknown benefit from a T&CM intervention that is commonly used^a^;
6. Inequalities of access or large unmet need;
7. High demand to integrate within conventional healthcare settings;
8. High costs relative to alternatives or vice versa;
9. Large system changes required for its introduction^a^; and
10. Conflict in choices between individual and societal perspectives^a^.

^a^Criteria outlined by the World Health Organisation for prioritising guideline development [7]

Insufficient evidence about any intervention or practice poses significant challenges for guideline developers. In the case of T&CM, failure to evaluate the field or even make a recommendation when there is insufficient evidence of efficacy may simply widen the gap between what practitioners and users of T&CM are doing, and what is considered best practice. Guideline developers should always attempt to make specific, informative recommendations about the use of T&CM [18–20]. As Petitti et al. state:

*“Decision makers do not have the luxury of waiting for certain evidence. Even though evidence is insufficient, the clinician must still provide advice, patients must make choices, and policy makers must establish policies”* [21].

Further guidance on how to use all available information and evidence for clinical decision making will help improve the utility of clinical guidelines that consider T&CM. Debate continues in the T&CM field and more generally around the appropriate use of evidence for evaluating interventions [22–24]. The objectives of this review, however, were to identify, appraise and synthesise nationally or internationally endorsed consensus statements for clinical guideline developers; and ‘interpret’ how these statements might apply to the field of T&CM, particularly in instances where there is low quality or inconsistent evidence regarding safety, efficacy or cost-effectiveness.

## Method

### Study design

A critical interpretive review of consensus statements for guideline developers was undertaken [25, 26]. The literature search and analysis of the consensus statements was an iterative process. A sampling frame was created where the identified consensus statements were coded and categorised into themes and subthemes on an electronic spreadsheet. The first author then summarised the findings for further discussion amongst the co-authors. Numerous iterations explored how best to categorise and interpret the themes until consensus was reached. Literature searching continued until there was data saturation (i.e. the point at which no new major themes emerged).

### Literature search & sampling

The consensus statements for guideline developers were identified using a similar approach to that used in the interpretive synthesis outlined by Dixon-Woods et.al [26]. The literature was searched from 7th April 2014 through to 10th October 2015. A systematic literature search was not conducted, because unlike Schünemann et.al, this review was not a content analysis where all consensus statements are identified to formulate a comprehensive list of items from these statements [27]. Instead, purposive sampling was used to identify statements published before the end-date of the search that clearly addressed the research objective. Consensus statements and publications were first identified through the authors’ expert knowledge of the topic. This was augmented by literature searches on Google Scholar and PubMed. Database searches using various sets of search terms (e.g. guidelines*, “Practice Guidelines as Topic/standards”[Mesh], Evidence-Based Medicine/methods[Mesh]) and search functions (e.g. customizing Article types) were abandoned because the results were either too broad or too narrow. Alternate search strategies were therefore employed, such as bibliographic searching of previously published systematic reviews [27], bibliographic cluster searching [28], and the use of ‘PubMed/Similar articles’ or ‘Google Scholar/Related articles’ functions.

### Inclusion & exclusion criteria

The authors defined a consensus statement as a document or similar resource (e.g. website) developed by an independent panel of experts that provided systematic guidance. In this instance, the guidance was on methodologies for formulating clinical guidelines or related health policies. Only statements endorsed by national or international authorities and published in English were included. Consensus statements on health policy making were also included since clinical guidelines are used not only to inform clinical decision making but to inform health service delivery and public health policies. Consensus statements describing how to appraise the quality of clinical guidelines were excluded as no new themes could be identified that were not already addressed in detail, including the rationale, in the statements on guideline development. Statements published from 1995 until the end of the search date in 2015 were included. For those with multiple iterations, only the most recent edition of a statement was included in the analysis.

### Data extraction & analysis

An interpretive approach was used to appraise and synthesise the information [25, 26]. This was an inductive process. As consensus statements and their related publications were identified, their content was reviewed for relevant themes applicable to the use, practice and context of T&CM (see Table 2), and the known shortcomings of existing clinical guidelines for T&CM [14–16, 29–31]. The statements were compared for similarities *(reciprocal translational analysis)* and contradictions *(refutational synthesis)*. Lines-of-arguments *(synthesising arguments)* were generated by integrating the content and themes identified in the individual statements. The aim was to identify overarching themes and constructs, and then *interpret* how they apply to T&CM.

**Table 2:** Contextual information about T&CM

1. T&CM often has different regulation than biomedicine because the interventions are considered relatively safe and/or there is a historical precedence of use spanning hundreds, if not thousands of years [29].
2. Clinical guidelines are often uninformative regarding the use of T&CM; and are lacking in transparency and consistency [14–16].
3. In instances of insufficient scientific evidence, information about clinical indications and safety; patient demand and preferences; or equity and costs may still be available [29].
4. The outcomes that users seek from T&CM do not always align with the outcomes evaluated in clinical trials [30].
5. The ongoing demand for T&CM suggests that factors other than scientific evidence are important and influence the decision making process of patients and practitioners [31].

## Results

Eight consensus statements for guideline developers met the inclusion criteria for in-depth review; this was the point at which data saturation was reached and no new major themes emerged. Three of the statements were international [7, 18–20, 32–63]; the remaining five were national statements from Australia [64–66], Germany [67],Scotland [68], US [21, 69–71] and UK [72–74].

The primary focus of the first seven statements (as listed in Table 3) was the development of clinical practice guidelines for the management or prevention of disease. These guidelines all used similar methodologies for systematically identifying and appraising the evidence of efficacy, safety and cost-effectiveness [7, 32, 64, 67, 68, 70, 72]. However, there were differences in the terminology and categories used to summarise the evidence and formulate recommendations. GRADE, AWMF and the NHMRC, for example, categorised the quality of the evidence and the strength of the recommendations [32, 66, 67]. Alternatively, NICE provided guidance on the wording of phrases to reflect the strength of the recommendations rather than using explicit grades or categories [72]. Both the USPSTF and SIGN included an option to make a non-specific recommendation for instances of genuine uncertainty [68, 70].

**Table 3:** Summary of consensus statements for guideline developers

1. Guidelines for W.H.O. guidelines [7]. A concise outline of the principles for guideline development, the statement emphasises the importance of considering local circumstances such as applicability of the research findings to the population and the feasibility and opportunity costs of implementing the guideline. Annex C provides a succinct yet comprehensive checklist for appraising treatment guidelines.
2. GRADE Grading of Recommendations Assessment, Development and Evaluation [18, 19, 32–44]. Endorsed by the Cochrane Collaboration and W.H.O., GRADE is an internationally recognised system for evaluating and comparing alternative interventions. Recommendations are graded based on the quality of evidence (i.e. study design, bias, consistency of the results, and precision of the overall estimates); risks and benefits; resources used; and values and preferencesResources include the online software GRADEproGDT for producing guidelines, Summary of Findings tables and Evidence to Decision Frameworks [45].
3. AWMF Regelwerk/Guidance Manual and Rules for Guideline Development [67]. The Association of the Scientific Medical Societies in Germany (AWMF) builds on the GRADE process. It also provides explicit guidance on how to structure consensus development, classify the strength of consensus and manage justified dissent to formulate a transparent set of recommendations. Accompanying statements included the DELBI 2.0 - Deutsche Leitlinien-Bewertungsinstrument/German Guideline Appraisal Instrument.
4. NHMRC: A guide to the development, evaluation and implementation of clinical practice guidelines [64]. The Australian National Health and Medical Council (NHMRC) guidelines along with seven accompanying handbooks were published from 1999 to 2003. Together they provide detailed information about reviewing and appraising clinical, economic and socioeconomic evidence; implementing and disseminating guidelines; and presenting the information to consumers. Guidelines on appraising evidence and grading recommendations were updated in 2009 and included a Body of Evidence Matrix [65, 66]. A ‘hierarchy of evidence’ constrains the grading of recommendations.
5. NICE: Developing NICE guidelines - The manual [72]. The National Institute for Health Care and Excellence (NICE) guidelines manual from the United Kingdom has evolved over time. Initially recommendations were rated according to a ‘hierarchy of evidence’ that reflected the quality of studies. This rating was replaced in 2006–7 with an approach that more closely aligns with GRADE. Differences to GRADE include modifications to the economic evidence profiles and the absence of ‘overall summary’ labels for the quality of evidence and strength of recommendations [74].
6. SIGN 50: A Guideline Developer’s Handbook [68]. The Scottish Intercollegiate Guidelines Network (SIGN), first published their handbook in 1999. It was modified over the years to reflect methodological changes in appraising the evidence and grading recommendations. A hierarchy of evidence was used from 2001 to 2012 that was discontinued in line with the SIGN’s adoption of the GRADE process in 2013.
7. U.S. Preventive Services Task Force (USPSTF) system [69]. The system is used to assess the quality, magnitude and confidence in the evidence about benefits and harms of an intervention and provide a graded recommendation [70]. An analytic structured causal framework of process and outcome is recommended to help guide the systematic evaluation of non-RCT study designs [71]. If the problem of insufficient evidence persists, the four “Domains of Information Pertinent to Clinical Decision making for Preventive Services” may be used [21].
8. SUPPORT Tools for evidence-informed health Policymaking (STP) [20, 46–63]. STP was written for people responsible for making decisions about health policies and programmes and for those who support these decision makers. The series addresses four broad areas: 1) Supporting evidence-informed policymaking 2) Identifying needs for research evidence in relation to three steps in policymaking processes, namely problem clarification, options framing, and implementation planning 3) Finding and assessing both systematic reviews and other types of evidence to inform these steps, and 4) Going from research evidence to decisions.

The eighth consensus statement included in this review, the SUPPORT guidelines [46] was the only statement aimed solely at evidence-informed health policy making, including decisions about healthcare services. The SUPPORT guidelines acknowledge controlled trials and systematic reviews as important, but in addition, they emphasise the value of obtaining other information and local evidence of modifying factors such as needs, values, costs and the availability of resources. Importantly, they also offer guidance on preparing and using policy briefs [53, 61].

Following a detailed analysis of the eight selected consensus statements and their related publications, five main themes emerged that were relevant to the challenges of developing T&CM recommendations and are particularly relevant when there is low quality, conflicting or inconsistent evidence. These were:

1. The importance of framing the question.
2. The limitations of an evidence hierarchy.
3. Methods for dealing with insufficient evidence.
4. Qualifying a recommendation.
5. Structured consensus development.

### Framing the question

All eight statements provided guidance about clarifying at the outset of the guideline development process the intended scope, questions, interventions and outcomes to be covered. The PICO process (Patient problem, Intervention, Comparison, Outcome) was often recommended to help formulate clinically relevant questions and patient-important outcomes were increasingly emphasised [36, 67, 68, 72].

Little guidance was provided however about methods for systematically identify potentially relevant interventions and selecting interventions for further in-depth systematic reviews. The WHO and NICE both provided guidance around choosing priority topics and interventions [7, 72]. This included interventions that were commonly used with unclear benefits and risks. The NICE 2012 edition was the only consensus statement that specifically mentioned high T&CM use by patients for managing the problem as a reason for inclusion, and the importance of searching databases relevant to T&CM evidence [73].

*“The effects of complementary and alternative therapies may be addressed in the guideline if such therapies are commonly used in the clinical area of interest. If commonly used complementary and alternative therapies are not to be covered in the guideline, this should be stated clearly in the scope.”* [73].

### Limitations of an evidence hierarchy

As the various recommendations for developing guidelines have been updated, there has been a move away from using a ‘hierarchy of evidence’ or ‘levels of evidence’ towards the GRADE approach to making recommendations. This is due to ongoing concerns that a hierarchy can inappropriately encourage guideline developers and policy makers to directly link study design to recommendation strength, or ignore lower levels of evidence that should also be included when grading the strength of the recommendation [74]. NICE ceased using an evidence hierarchy in 2007–8, followed by SIGN in 2012; notwithstanding, the 2014 edition of SIGN still refers to levels of evidence in the “Example pages from an evidence table” [68].

The Australian National Health and Medical Research Council (NHMRC) guideline was the only included consensus statement that continued to use an evidence hierarchy as a direct constrainer on the strength of the recommendations [66]. According to these guidelines, the strongest recommendations, an ‘A’ or ‘B’, can only be made if the evidence quality is also graded as an ‘A’ or ‘B’. The NHMRC does acknowledge that questions about safety – especially for uncommon adverse events from treatments or harms from diagnostic testing – are unlikely to be answered through randomised controlled trials and in such cases, consideration of lower levels of evidence are permitted.

### Dealing with insufficient evidence

GRADE and the USPSTF statements provided the most specific advice on how to manage the challenges of insufficient, high quality evidence. The SUPPORT statement most clearly emphasised that inconclusive results or lack of research should not be misinterpreted as evidence of no effect. Despite insufficient evidence about effectiveness, informed decisions can still be made about interventions that are potentially harmful or when the potential benefits are not worth the cost [20].

In situations of low quality evidence for an intervention and a lack of confidence in the effect estimates of the risks and benefits, the GRADE statements outlined five instances when a strong recommendation could still be made [18]. Table 4 is a modification of the GRADE guidelines where the original non-T&CM examples are replaced with examples pertinent to T&CM.

**Table 4:** The application of the GRADE *“Paradigmatic situations in which a strong recommendation may be warranted despite low or very low confidence in effect estimates”* for T&CM^a^

1. When low quality evidence suggests benefit in a life-threatening situation (evidence regarding harms can be low or high)
*e.g. Very low quality evidence for intravenous silibinin for life-threatening amatoxin mushroom poisoning* [91, 92], or *intravenous ascorbate for life-threatening viral infections* [93, 94]. Recommend to use if there are no alternate proven interventions.
2. When low quality evidence suggests benefit and high quality evidence suggests harm or a very high cost
* e.g. Low quality evidence that intravenous ascorbate improves quality of life and reduces chemotherapy toxicity in cancer treatment, and high quality of evidence of very high costs compared to oral ascorbate* [95]. If there are significant opportunity costs to the patient, recommend not to use intravenous.
3. When low quality evidence suggests equivalence of two alternatives, but high quality evidence of less harm for one of the competing alternatives
*e.g. Low quality evidence of equivalence of glucosamine and non-steroidal anti-inflammatory drugs (NSAIDS) for the long-term symptomatic management of osteoarthritis; but high quality evidence of increased gastrointestinal and cardiovascular risks for NSAIDs only* [96, 97]. Recommend glucosamine as first-line treatment, especially for patients with a higher risk of complications from NSAIDs.
4. When high quality evidence suggests equivalence of two alternatives and low quality evidence suggests harm in one alternative
* e.g. High quality equivalence of certain proprietary extracts of St. John’s Wort and selective serotonin reuptake inhibitors (SSRIs) for depression; and low quality evidence of a higher risk of suicide with SSRIs* [98–100]. If these certain proprietary St. John’s Wort extracts are available and affordable, then recommend as first-line treatment before an SSRI.
5. When high quality evidence suggests modest benefits and low/very low quality evidence suggests the possibility of catastrophic harm
*e.g. High quality evidence of modest benefit from peri-operative use of fish oil for elective Coronary Artery Bypass Surgery; and low quality evidence for catastrophic haemorrhage* [80–82]. Recommend not to cancel CAB surgery if the patient has taken fish oil pre-operatively.

^a^Modified from GRADE where non-TC&M examples were presented [18]

In the case of inconclusive or absent evidence from randomised controlled trials (RCTs) and meta-analyses, the USPSTF proposed several instances where the assemblage of non-RCT evidence would be admissible in clinical guidelines. The first is where an intervention is potentially effective, there is a large burden of disease and there is no research investigating the direct effects of the intervention on the health outcome [71]. In this instance, a Generic Analytic Framework (GAF) could be constructed to answer a sequence of key questions that form a chain of evidence about benefits and risks [71]. Recommendations can then be formulated based on indirect evidence linking the intervention to the outcome. For example, an intervention that demonstrates a reduction in the incidence rate of ischaemic heart disease (IHD) is direct evidence. When the same intervention has only demonstrated an ability to lower a person’s weight, other research must be linked to provide indirect evidence that losing weight can reduce known IHD risk factors and the likelihood of developing IHD. The safety, acceptability and costs of the intervention are also considered. The USPSTF further recognises that different types and quality of evidence will be required to link the evidence.

The second instance proposed by the USPSTF for the assemblage of non-RCT evidence is when the intervention is not amenable to being evaluated under RCT conditions [21]. Examples given included various behavioural interventions for substance abuse where either there is no appropriate control for blinding, or it is impossible to provide the treatment fidelity required for an RCT because it would eliminate the individualised, adaptive treatment approach that is needed for success.

For instances when despite using the above two suggestions there remains insufficient evidence, the USPSTF recommends structuring information around the following four domains to explicitly present data for decision makers:

1. Burden of suffering – the incidence and prevalence of a condition; the degree of personal, family and community suffering; and the burden to families, society and health care systems.
2. Potential harm – the immediate and long-term harms to individuals and patients from delivering an intervention or service and from alternatives, including the potential harms associated with doing nothing.
3. Cost – the direct monetary costs of a service or intervention; the opportunity costs, such as the time, money and resources that would be diverted to provide an intervention with less evidence or acceptability to patients; and the costs of decommissioning the intervention should it then prove to be ineffective.
4. Current practice – the potential negative consequences (including legal) of providing a novel, less widely used service or intervention compared to those commonly in place; and the extra resources that will be needed to change ingrained practice [21]. (In the case of T&CM, this question might also be extended to consider the consequences of removing or restricting access to commonly used interventions).

Both the USPSTF and SIGN included a category for recommending the use of interventions in the research setting only [68, 70]. The USPSTF stated that only-in-research recommendations should be reserved for promising interventions where there is the potential to cause significant harm or there are high costs [70]. The latter includes interventions where there is a large component of fixed costs that cannot be retrieved if the intervention is withdrawn [21]. Conversely, GRADE did not provide an only-in-research category. Such a recommendation is possible however, if the following three conditions are met:

1. There is genuine uncertainty from the existing evidence;
2. Further research is very likely to remove or reduce this uncertainty; and
3. The cost of further research is deemed to be good value [19].

### Qualifying a recommendation

There was general consensus across all statements that an evidence-based guideline is unhelpful if it fails to provide information about modifying factors. Contextual information about the burden of disease and available interventions; generalisability and applicability to population groups; direct and indirect costs; demand, accessibility and equity; and the values and preferences of patients and providers is increasingly being used to help select interventions, identify relevant outcomes for appraising the evidence, provide information about benefits and risks, and to qualify recommendations [4–7, 72].

High quality evidence in support of these modifying factors may justify upgrading or downgrading a recommendation [18]. For example, patients may consider the most effective intervention to be unacceptable due to their personal tolerance for risk, or other personal values such as a preference for natural therapies. In the case of healthcare providers and policy makers, equity, costs and current service provision are likely to be influencing factors. An intervention with small clinical impact (effect size) that is widely used or readily available, may be preferred to an intervention with large clinical impact that is significantly more expensive or requires substantial system changes to integrate into practice. That patients or policy makers make different choices based on preferences, values and costs, are reasons why an intervention with high quality scientific evidence of efficacy may still be downgraded to a weak recommendation and vice versa [18, 19].

The NHMRC proposed a system that includes the grading of modifying factors [65, 66]. The NHMRC Evidence Matrix grades the evidence for safety, efficacy, cost-effectiveness, consistency of results, clinical impact, the generalisability of the evidence and its applicability to the Australian healthcare setting. Evidence about other important modifying factors however, such as patient and provider preferences were not included.

Only-in-research recommendations also require qualification. GRADE for example actively discouraged blanket statements recommending further scientific research [18]. Instead, such recommendations should include justification of the need for further research and detail the research questions with particular attention given to patient-important outcomes [19, 21].

### Structured consensus development

All eight statements in this review emphasised that membership of a guideline development committee should represent the relevant stakeholders. AWMF 2.0 was the only statement however to recommend and outline scientifically sound formal consensus methods to promote transparency and resolve conflicts arising from differences of opinion [67]. Given the complexity of the decision-making process that necessitates sourcing and appraising all the information, non-objective personal and professional biases are likely to emerge when selecting interventions and outcomes, appraising modifying factors, and formulating recommendations. Standardised methods such as the Nominal Group Process, the Structured Consensus Conference and the Delphi Technique were recommended. The ultimate aim is to improve the transparency, quality, reproducibility and acceptability of the recommendations [67].

## Discussion

This is first known review to synthesise the content and themes of national and international consensus statements for developing clinical and health policy guidelines and to interpret these through the lens of T&CM. Given the influence of the evidence-based medicine movement on clinical practice, education and health policy, it is not surprising that the majority of statements reviewed in this paper provided detailed guidance on how to systematically identify and appraise evidence of efficacy [27]. The limitations of using a didactic 'recipe book' approach when formulating recommendations was increasingly being recognised; particularly the limitations of using an evidence hierarchy and the importance of modifying factors [24, 74]. The USPSTF statements provided the clearest guidance and strategies for dealing with insufficient evidence.

Notwithstanding alternate, more pragmatic approaches to evidence appraisal such as those proposed by the USPSTF, the paucity and heterogeneity of scientific evidence for many T&CM interventions remains a significant challenge to guideline developers. It is important not to imply that inconsistent evidence or an absence of evidence means there is evidence of no effect [20]. In these instances the general consensus was that guidelines should still attempt to make specific recommendations or at least offer some information to help guide decisions [18, 20, 21]. Table 4 lists the paradigmatic circumstances proposed by GRADE where a strong recommendation could be made despite low quality evidence [18]. Guideline developers should be mindful of these instances and not automatically default to a recommendation not to use an intervention based solely on low quality scientific evidence regarding efficacy [18, 21].

The early use of an evidence hierarchy that places the RCT and meta-analyses at the pinnacle may help explain the ad-hoc inclusion and appraisal of T&CM in clinical guidelines, especially older guidelines [16]. If higher levels of evidence are lacking and lower levels of evidence are discounted with no qualifying statements, gaps in the evidence review are likely to occur and an intervention overlooked [74]. The guidelines may then default to non-informative statements and recommendations, as was found to be the case in the reviews of UK clinical guidelines [16]. Consistent with international standards, bodies such as the Australian NHMRC should cease endorsing the use of ‘levels of evidence’ as a direct constrainer of ensuing recommendations and instead make greater use of qualifying statements that consider important modifying factors, including those relevant to patients and practitioners.

The USPSTF suggested a number of instances when the double-blind RCT is not the most appropriate study design [18–21]. Although the specific components of a T&CM intervention may be amenable to assessment using an RCT design, there are many instances where this is not appropriate [75]. For example, for some T&CM interventions, finding an adequate control may be difficult or impossible; and for others, treatment fidelity would be lost due to the individualised, multifaceted approach of the therapy or the complexity of the study outcomes that are multiple and holistic, with some being immediate and others delayed [76, 77].

A potential T&CM example for the assemblage of admissible non-RCT evidence is acupuncture for depression [21, 71]. Depression is an illness where there is a large burden of disease and there is growing pragmatic evidence of effectiveness, but weak or conflicting evidence from double-blind RCTs about the efficacy of acupuncture [78]. The challenge with finding a suitable control for acupuncture, as well as the individualised nature of the intervention, may explain the mixed results from efficacy (explanatory) trials compared to the more consistent positive results from effectiveness (pragmatic) trials [79]. In cases such as this, it may even be justified to give a lower weighting to the quality score of study designs that use a non-individualised treatment protocol or an inappropriate control.

Due to the paucity of a large body of high quality evidence regarding efficacy for many T&CM interventions, a common recommendation from systematic reviews and clinical guidelines is to make a general call for further research. This is unhelpful to clinicians and patients who need immediate guidance and should only be made if the research is warranted [19, 21]. A recommendation for further research should only be made for interventions where there is true uncertainty about risks and benefits; especially if there are large direct costs or opportunity costs, or there is the potential for large benefit from wider, more equitable use [20]. For example, along with the strong recommendation not to cancel coronary artery bypass surgery if a patient has taken fish oil preoperatively (see Table 4: example 5) [80–82]; recommendations for further research are justified. Treatment duration and doses of EPA and DHA requires further clarification. There is potential for different populations to disproportionally benefit (e.g. socioeconomic, ethnic, or other groups with specific cardiovascular risk factors). Economic evaluations are also warranted since the cost of fish oil, even with only modest clinical benefit, may be cost-effective compared to the cost of surgical complications; and health inequalities are a concern since patients commonly pay 100% out-of-pocket to use fish oil.

Including modifying factors when qualifying recommendations enhances their relevance to different clinical scenarios and populations [18, 21]. The diverse and potentially conflicting information about efficacy and relevant modifying factors is particularly challenging for guideline developers. Modifying factors can be used for example to upgrade or downgrade the strength of a recommendation independent of the quality of the evidence [18, 19]. This point is particularly relevant to T&CM interventions where there is insufficient high quality scientific evidence regarding efficacy and effectiveness. In these instances, high quality evidence may still be available about the burden of disease; risks of alternate therapies; direct and indirect costs; demand, access, affordability and equity; generalisability and applicability of the intervention to specific population groups; patient and provider values and preferences; and implementation and feasibility [7–10, 27, 32, 83]. It is therefore inappropriate to limit systematic literature reviews for informing guideline development only to questions about safety, efficacy and cost-effectiveness.

To elaborate, there is high quality evidence that hormone replacement therapy (HRT) is effective for managing menopausal symptoms; however, there is also high quality evidence about the risks of HRT. [84, 85] By contrast, there is conflicting evidence about the efficacy of Black Cohosh for managing menopausal symptoms and very low quality evidence questioning its safety [86, 87]. There is also high quality evidence that some women would prefer to use potentially less effective natural approaches to manage these non-life-threatening symptoms, of which herbs such as Black Cohosh are amongst the most popular choices [88–90]. Although many clinical guidelines qualify the recommendation to use HRT with a statement about assessing the risks and benefits of hormone use for an individual patient, most fail to make any qualifying statements about known patient preferences to use T&CM, its comparative safety, and the direct costs and opportunity costs of first trialling a potentially less efficacious intervention [90].

The inconsistencies regarding the inclusion of T&CM and recommendations made about their use in clinical guidelines calls for a more transparent and systematic approach to guideline development. Even formal methods for consensus development such as those outlined in AWMF [67] will be prone to bias if the expert committee for example, brainstorms or uses other non-systematic methods to select comparison interventions. The scope, interventions and outcomes will then likely reflect the experience and knowledge of the members of the committee, or other biases such as only considering interventions that are thought to have high quality evidence and worthy of consideration. As WHO and NICE highlighted, amongst other reasons (see Table 1) T&CM should be considered if they are commonly used in the clinical context [7, 73], irrespective of the quality of evidence about their benefits and risks [7].

## Conclusion & recommendations

This interpretive review has considered, for the first time, the usefulness of directives for developing guidelines and recommendations regarding T&CM practice and policy. Like many areas of healthcare, insufficient evidence about efficacy poses significant challenges to guideline developers, which in the field of T&CM has contributed towards insufficient and inconsistent recommendations. The emerging and heterogeneous evidence-base for many T&CM interventions necessitates a range of methodologies to ensure the systematic selection of interventions and consideration of modifying factors when formulating and qualifying recommendations. In light of these issues and the high demand for T&CM, we behove guideline developers to consider T&CM from a number of perspectives when appraising the evidence, and to make clinically useful and specific recommendations regarding their use.

Specifically, guideline developers should cease endorsing an evidence hierarchy as a direct constrainer of recommendations. Strict use of the levels of evidence runs the risk of inappropriately linking the quality of the evidence for efficacy directly to the strength of the recommendation, whilst ignoring admissible non-RCT evidence and important modifying factors. In instances of very low quality or equivocal evidence of efficacy, guideline developers must consider the paradigmatic situations where nonetheless a strong recommendation can be made. Failing this, broader contextual information is often available for T&CM even when there is low quality scientific evidence regarding efficacy. Information about modifying factors should be presented to facilitate informed decision making and improve clinical relevance. Finally, greater attention must be given to adopting a systematic and transparent approach to the entire development process, including the selection of comparative interventions and patient relevant outcomes. The uptake of these recommendations is expected to result in higher quality clinical guidelines that offer greater assistance to those seeking answers about the appropriate use of T&CM.
